# Expression of ABCG2 (BCRP) in mouse models with enhanced erythropoiesis

**DOI:** 10.3389/fphar.2014.00135

**Published:** 2014-06-04

**Authors:** Gladys O. Latunde-Dada, Abas H. Laftah, Patarabutr Masaratana, Andrew T. McKie, Robert J. Simpson

**Affiliations:** ^1^Diabetes and Nutritional Sciences Division, School of Medicine, King's College LondonLondon, UK; ^2^Vascular Sciences Unit, Imperial Centre for Translational and Experimental Medicine, Imperial College, NHLILondon, UK; ^3^Department of Biochemistry, Faculty of Medicine Siriraj Hospital, Mahidol UniversityThailand

**Keywords:** *Abcg2*, *bcrp*, hypoxia, phenylhydrazine, iron

## Abstract

Haem is a structural component of numerous cellular proteins which contributes significantly to iron metabolic processes in mammals but its toxicity demands that cellular levels must be tightly regulated. Breast Cancer Resistance Protein (BCRP/ABCG2), an ATP Binding Cassette G-member protein has been shown to possess porphyrin/haem efflux function. The current study evaluated the expression and regulation of *Abcg2* mRNA and protein levels in mouse tissues involved in erythropoiesis. *Abcg2* mRNA expression was enhanced in bone marrow hemopoietic progenitor cells from mice that were treated with phenylhydrazine (PHZ). *Abcg2* mRNA expression was increased particularly in the extramedullary haematopoietic tissues from all the mice models with enhanced erythropoiesis. Haem oxygenase (*ho1*) levels tended to increase in the liver of mice with enhanced erythropoiesis and gene expression patterns differed from those observed in the spleen. Efflux of haem biosynthetic metabolites might be dependent on the relative abundance of *Abcg2* or *ho1* during erythropoiesis. *Abcg2* appears to act principally as a safety valve regulating porphyrin levels during the early stages of erythropoiesis and its role in systemic haem metabolism and erythrophagocytosis, in particular, awaits further clarification.

## Introduction

Iron turnover in mammals is largely explained by the production (biosynthesis) and breakdown (biodegradation) of erythrocytes. Consequently iron fluxes during erythropoiesis and erythrophagocytosis must be balanced and this is mainly achieved by influences of hepcidin in the maintenance of iron homeostasis. Three essential substrates, iron, porphyrin, and haem as well as their combination, are essential for erythropoiesis but are potentially toxic if allowed to accumulate. Iron, haem, and photosensitized porphyrin are pro-oxidants and they can cause damage to DNA, proteins and cell membrane systems. Availability of iron, a limiting nutrient, and the *de novo* synthesis of haem and porphyrin are therefore strictly coordinated and channeled to avert the accumulation of excess toxic metabolites. Recently the identification of two haem efflux proteins, Breast Cancer Resistant Protein (BCRP or ABCG2) and Feline Leukaemia Virus C Receptor (FLVCR) has defined important mechanisms that protect cells and tissues against noxious free haem (Quigley et al., 2004; Krishnamurthy and Schuetz, 2005). Abcg2 is a half ATP-Binding Cassette (ABC) G-member transporter with a nucleotide binding domain (NBD) at its amino terminus and a transmembrane domain at its carboxyl end (Krishnamurthy and Schuetz, 2006). It functions conventionally as a plasma membrane protein extruding endogenous and exogenous toxic xenobiotics from the intestine, liver, placenta, and the blood/brain barrier. This transporter, in some instances, confers resistance to anticancer drugs. The broad substrate spectrum of abcg2 are porphyrin metabolites (Suzinges-Mandon et al., 2010). Specifically, *Abcg*2 was shown as a porphyrin efflux protein which functions to rid erythroid cells of excess porphyrins particularly under hypoxic conditions (Krishnamurthy et al., 2004) and thereby protects haematopoietic cells from the potential toxicity of excess free porphyrin compounds. As hypoxia could vary in magnitude depending on the duration and the type of anaemia, the detoxification function of *abcg2* might be pertinent in the various other tissues that handle large quantities of haem and its degradation products e.g., liver.

At the cellular level, haem oxygenase 1 (ho1) catalyses the rate limiting step in the degradation of haem into iron, carbon monoxide, and biliverdin, which is reduced by biliverdin reductase to bilirubin. These by-products also constitute potential substrates for abcg2, and have recently been shown to have antioxidant, anti-inflammatory, and cytoprotective functions (Bilban et al., 2008; Soares and Bach, 2009). Excess haem is presumed to be channeled into the lysosome for catabolism or might be effluxed into circulation where it binds to hemopexin (Tolosano et al., 2010). Haem and its metabolites may be substantially and strategically trafficked or otherwise degraded in a coordinated and regulated manner under different modulators of iron metabolism. Moreover, abcg2 might play a vital role in the transport of haem and porphyrin compounds during erythropoiesis. This study investigates the expression and regulation of *abcg2* and *ho1* in tissues of mice with enhanced erythropoiesis.

## Materials and methods

### Animals and tissue collection

CD1 mice were used for all experiments except the hypotransferrinaemic (Hpx) mice that are of Balb/c background. Mice were placed in a hypobaric chamber at 0.5 atm for 72 h to effect hypoxia. Controls were kept at room air pressure. Dietary iron deficiency was induced by feeding 4-week old CD1 male mice with a low-iron diet (Formula TD. 80396, elemental iron concentration: 3–6 mg/kg or control diet containing 48 mg/kg iron, TD. 80394; Harlan-Teklad; Madison, WI, USA) for 3 weeks. Enhanced erythrocyte turnover was induced by intraperitoneal injections of neutralized phenylhydrazine (PHZ) at 60 mg/kg body weight; (Sigma-Aldrich, UK) on 2 consecutive days. All mice were maintained on standard commercial diet (Rodent Maintenance diet; RM1, Special Diet Services, UK) feed and water *ad libitum*. Mice were sacrificed after anaesthesia and neck dislocation, after which tissues were collected. Femurs were excised and flushed with Dulbecco's minimum essential medium (DMEM; Sigma-Aldrich, UK), to collect the hemopoeitic progenitor cells from the bone marrow of control and PHZ-treated CD1 mice. All procedures were approved and conducted in accordance with the UK. Animals (Scientific Procedures Act, 1986).

### Cell culture

#### RT-PCR

Total RNA was extracted from tissue samples using Trizol reagent (Invitrogen, UK) according to manufacturer's instructions. Quantitative RT-PCR was carried out using an ABI Prism 7000 detection system in a two-step protocol with SYBR Green (ABI, Life Technologies, UK). The efficacy of the amplification was confirmed by a melting curve analysis and gel electrophoresis to confirm the presence of a single product.

Quantitative measurement of each gene was derived from a standard curve constructed from known amounts of PCR product. The results were calculated by the ΔC_*t*_ method that expresses the difference in threshold for the target gene relative to that of 18S RNA. Sequences of primers used, forward, and reverse, respectively, are as follow:

Mouse abcg2 5′-TCGCAGAAGGAGATGTGTTGAG-3′
5′-CCAGAATAGCATTAAGGCCAGG-3′
Mouse tfr1, 5′-CATGGTGACCATAGTGCACTCA-3′
5′-AGCATGGACCAGTTTACCAGAA-3′
Mouse ho1 5′-CAAGGAGGTACACATCCAAGCC-3′
5′-TACAAGGAAGCCATCACCAGCT-3′
Mouse 18s 5′-GAATTCCCAGTAAGTGCGGG-3′
5′-GGGCAGGGACTTAATCAACG-3′
Human 18s 5′-AACTTTCGATGGTAGTCGCCG-3′
5′-CCTTGGATGTGGTAGCCGTTT-3′

#### Western blot analysis

Spleen tissue was homogenized (in a buffer containing 50 mM mannitol, 2 mM Hepes, 0·5 mM PMSF and pH 7·2) with an Ultra Turrax (IKA, Staufen, Germany) homogenizer in (3 × 30 s pulses at full speed). The homogenate was centrifuged at 1500 g for 5 min and the supernatant was centrifuged for 1 h at 15,000 g to obtain the crude membrane fraction. Protein concentration was determined using Bio-Rad reagents (Bio-Rad, Laboratories, Hercules, CA, USA). Fifty (50) μg of membrane extracts were loaded onto a 12% gel in a SDS-PAGE. The proteins separated were then transferred to Hybond ECL-nitrocellulose membrane (Amersham Biosciences, Bucks, UK) using a Bio-Rad semidry transfer apparatus (Trans-Blot^R^ SD Semi-Dry Transfer Cell; Bio-Rad, UK). Membranes were blocked with 5% milk for 1 h and probed with ABCG2 BXP-53 monoclonal (Santa Cruz Biotechnology, USA), and, β-actin (Sigma, UK) antibodies diluted in 0.01% milk in TBS. Cross-reactivity was observed with peroxidase-linked anti-IgG by using SuperSignal West Pico (Thermo Scientific, USA).

#### Statistical analysis

All values are expressed as mean ± s.e.m. Statistical differences between means were calculated with Microsoft Excel 6.0 (Microsoft, Seattle, WA, USA) by using the Student *t*-test correcting for differences in sample variance. When multiple comparisons were necessary, One-Way analysis of variance (ANOVA) was performed using SPSS 14 (SPSS Inc, Chicago, US) with Tukey's *post-hoc* test.

## Results

**Figure 1:**
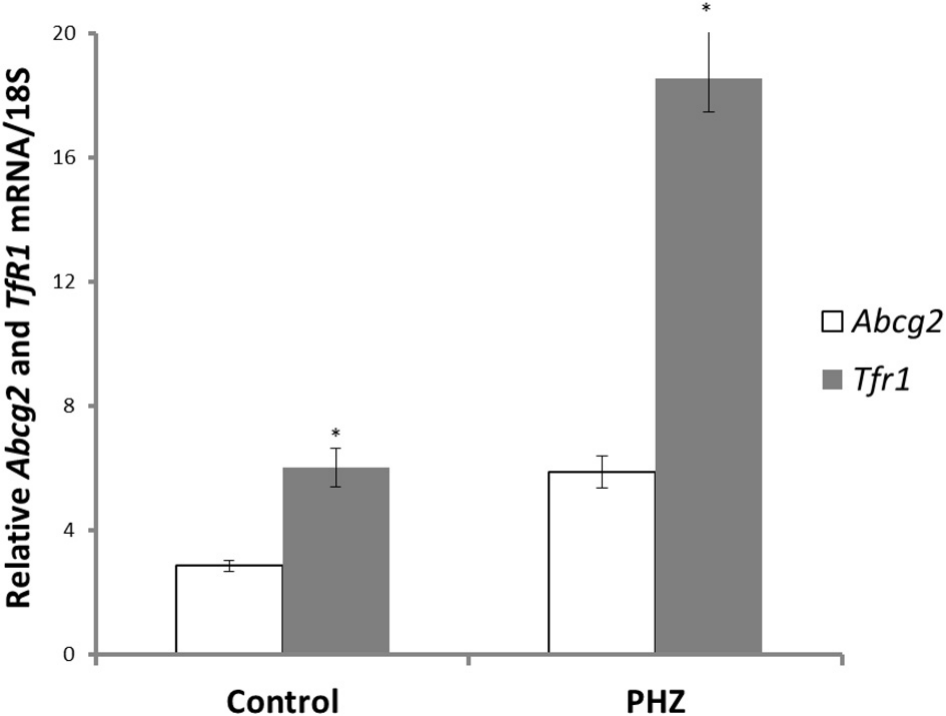
***Abcg2* and *TfR1* mRNA levels in bone marrow precursor cells of CD1 mice**. Erythropoiesis was induced with phenylhydrazine (PHZ) administration (60 mg/kg) over 2 days. Relative mRNA expression was obtained by normalizing mRNA levels to 18S mRNA. Analysis by Students' *T*-test showed significant effects of *abcg2* (*P* < 0.01), and *TfR1* (*P* < 0.005) over the control samples.

**Figure 2:**
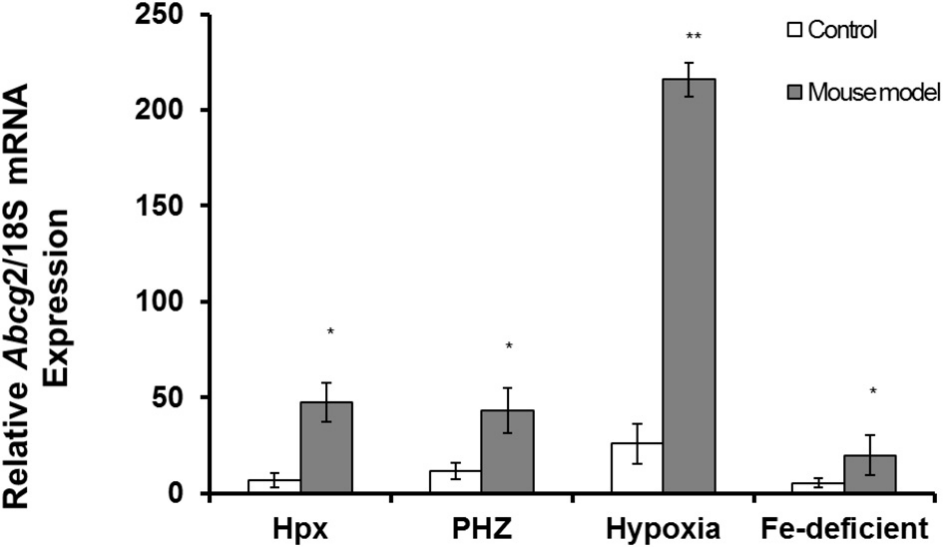
**Quantitative Real-Time PCR of *Abcg2* mRNA levels in spleen of mouse models with enhanced erythropoiesis**. *Abcg2* mRNA levels in the spleen of Hpx, PHZ-treated, hypoxic, and iron-deficient mice. Data are mean ± s.e.m. of four observations in each group and are expressed as a ratio of 18S mRNA in arbitrary units. Analysis by Students' *T-test* showed significant effects of *abcg2* mRNA in Hpx (*P* < 0.01), PHZ (*P* < 0.01), hypoxia (*P* < 0.05), and iron-deficient spleen tissues, (*P* < 0.05) over the control samples.

**Figure 3:**
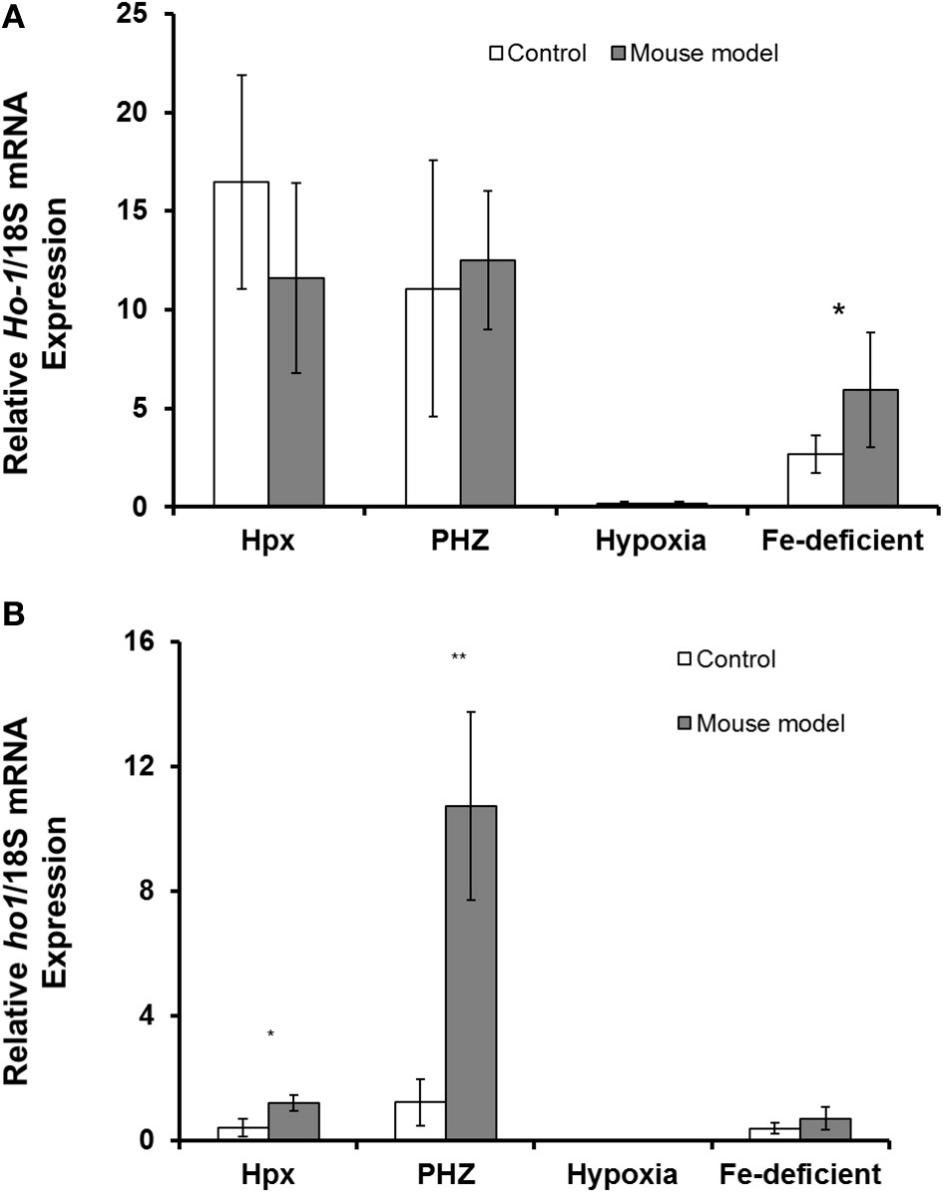
**Quantitative Real-Time PCR of *Ho1* mRNA levels in spleen and liver of mouse models with enhanced erythropoiesis. (A)** *Ho1* mRNA levels in the spleen of Hpx, PHZ-treated, hypoxic, and iron deficient mice. Analysis by Students' *T*-test showed significant effects of *Ho1* only in iron-deficient spleen tissues, (*P* < 0.01) over the control samples. **(B)** *Ho1* mRNA expression in the liver of Hpx, PHZ-treated, hypoxic, and iron deficient mice. Analysis by Students' *T*-test showed significant effects of *Ho-1* mRNA in Hpx (*P* < 0.05), and PHZ liver tissues, (*P* < 0.01) over the control samples. Data are mean ± s.e.m. of four observations in each group and are expressed as a ratio of 18S mRNA in arbitrary units.

**Figure 4:**
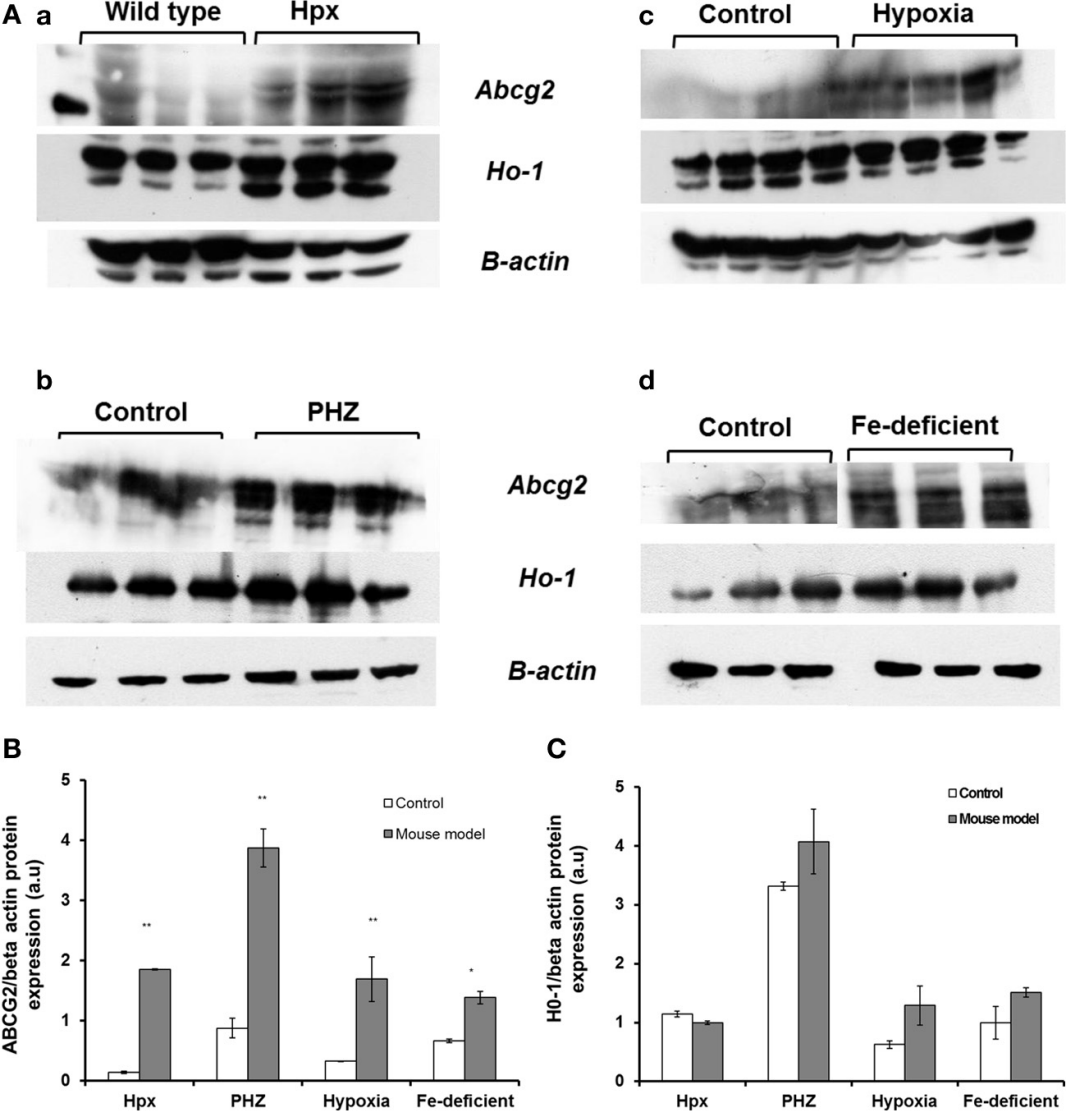
**Western blot analysis of Abcg2 protein and Ho1 levels in the spleen samples from Hpx, PHZ-treated, hypoxic and iron-deficient mice. (A)** Abcg2 protein levels in mouse spleen samples that were homogenized and subjected to Western blotting as described under methods. **(B)** Abcg2 protein levels in the spleen tissues. Densitometries of the bands (upper) were quantified with Image J software and data were normalized with the levels of β-actin protein in the samples. **(C)** Ho1 protein levels in the spleen tissues from mice. Densitometric data are presented as the mean ± s.e.m. of four experiments in each group. ^*^P < 0.05, ^**^P < 0.001.

*Abcg2* and *tfr1* mRNA expression were enhanced in bone marrow cells from mice treated with PHZ (Figure 1). Increased erythropoietic activity, which typifies Hpx, PHZ-treated, hypoxic, and iron-deficient mice, was associated with significant increases in *abcg2* mRNA expression in the spleen tissues from the mice (Figure 2). While changes in *ho1* mRNA expression in spleen tissues varied widely with the mice models (Figure 3A), hepatic *ho1* mRNA was induced significantly in Hpx and PHZ-treated mice (Figure 3B) instead. Enhancement of abcg2 protein expression in the spleen from mice with enhanced erythropoiesis correlated with the pattern of increased mRNA expression (Figures 4A,B). There was a slight, but observable, increase in ho1 protein levels in spleen tissues from PHZ-treated, hypoxic, and iron-deficient mice (Figures 4A,C).

**Figure 5:**
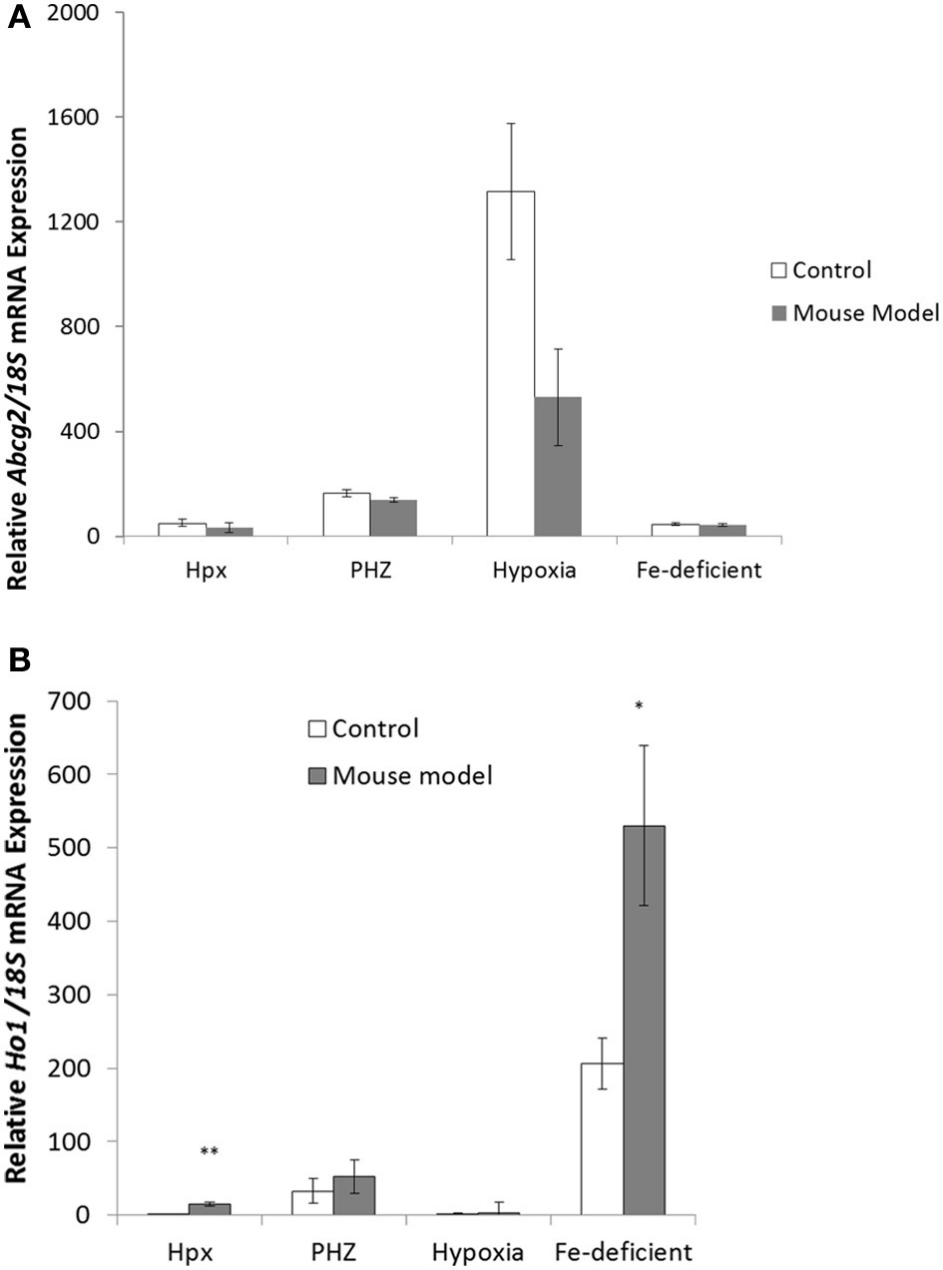
**Quantitative Real-Time PCR of *Abcg2* and *Ho1* mRNA levels in the kidney tissue samples from mouse models with enhanced erythropoiesis. (A)** *Abcg2* mRNA levels in the kidney of Hpx, PHZ-treated, hypoxic, and iron-deficient mice. Data are mean ± s.e.m. of four observations in each group and are expressed as a ratio of 18S mRNA in arbitrary units. **(B)** *Ho1* mRNA levels in the kidney of Hpx, PHZ-treated, hypoxic, and iron-deficient mice. Data are mean ± s.e.m. of four observations in each group and are expressed as a ratio of 18S mRNA in arbitrary units. Analysis by Students' *T*-test showed significant effects of *HO-1* mRNA levels in Hpx (*P* < 0.01), and iron-deficient spleen tissues, (*P* < 0.01) over the control samples.

*Abcg2* mRNA expression showed decreases in mouse model with enhanced erythropoiesis in kidney tissues (Figure 5A) but the response were not significantly different. *Ho*1 mRNA expression was significantly enhanced in kidney tissues from Hpx and iron-deficient mouse models (Figure 5B). Only modest increases in *ho1* mRNA expression levels were observed in kidney tissues from PHZ-treated and hypoxic mouse models.

## Discussion

Abcg2, an efflux protein with broad substrate specificity, is characteristically localized and expressed in epithelial membranes of barrier tissues such as the intestine, placenta, liver and the brain. The regulation of cellular haem and porphyrin levels by *abcg*2 plays a pivotal physiological function in erythropoiesis and iron metabolism in mammals. *Abcg*2 mRNA expression coincides with enhanced stimulation of haemoglobin biosynthesis (Zhou et al., 2005), during differentiation between BFU-E and CFU-E. Globin transcription and iron uptake are enhanced and haem concentration is critical for the initiation of haemoglobin synthesis (Krishnamurthy and Schuetz, 2005). This fact might be the reason for the increase in *abcg2* mRNA expression in the hemopoeitic precursor cells in mice that were administered with PHZ (Figure 1). As haemoglobinization increases with the progression of erythropoiesis, proerythrocytes presumably express low levels of *ho1* and are highly sensitive to low levels of cytosolic haem. *Abcg2* prevents porphyrin toxicity especially under hypoxic conditions, during the mid to the distal stages of erythropoiesis. Flvcr1 also effluxes excess haem during erythropoiesis and erythrophagocytosis (Keel et al., 2008; Chiabrando et al., 2012; Byon et al., 2013). Typical of promoters devoid of the TATA box, *abcg2* promoter has multiple transcription sites (Lin et al., 2001). Zong et al. (2006) showed multiple transcription start sites from several leader exons in *abcg2* gene. Moreover, these promoter regions are enriched with several transcription factors binding sites (Ishikawa et al., 2013). Furthermore, *abcg2* promoter is regulated by tissue specific transcription factors such as GATA 2 (Minegishi et al., 1998). Consequently, *abcg2* gene regulation is varied and multifarious in tissues and during diverse physiological processes.

Similarly, *abcg2* mRNA expression (Figure 2) and protein levels (Figures 4A,B) were consistently up-regulated in the spleen of mice with enhanced erythropoiesis. *Abcg*2 and, more recently *flvcr1* (Vinchi et al., 2014), have been reported to regulate haem levels in cells during erythropoiesis. Evidence from literature suggests a reciprocal pattern in *flvcr* vs. *abcg2* expression as differentiation progresses (Keller et al., 2006). *Flvcr* null mice are non-viable and this confirms a lack of functional overlap with *abcg2*. *Flvcr* is presumed to efflux haem into circulation from macrophages (Keel et al., 2008) and possibly functions specifically during erythrophagocytosis. The latter report implied that a proportion of haem from phagocytosed, senescent red blood cells in macrophages is not degraded but effluxed into circulation by *flvcr*. This haem “cargo” could subsequently be bound by hemopexin for delivery to the liver. It has recently been shown that hemopexin is obligatory to the functional haem efflux capability of *flvcr* (Yang et al., 2010). *Flvcr*, in contrast to some other members of the major facilitator superfamily, is a unidirectional, vectorial transporter.

Similarly, splenic expression of *abcg2* might also correlate with its role in detoxification processes. Haem, apart from being endogenously synthesized ubiquitously by erythroid and non-erythroid tissues, is also derived from haemolysis and haemorrhage (Reeder et al., 2002). While PHZ-treated mice are characterized by increased haemolysis and destruction of red blood cells, *ho1* mRNA expression was significantly enhanced in the liver rather than in the spleen of these mice (Figures 3A,B). The reasons for variation in the basal levels of *ho1* in the different mice models used in this study remain, however, both puzzling and unclear. The regulation of haem thus favors the depletion of unbound forms (Vinchi et al., 2008) to ameliorate toxicity in cellular and subcellular loci of visceral and peripheral tissues. It is well documented that *ho1* expression is highly inducible in the presence of haem (Schwarzer et al., 1999; Desbuards et al., 2009). Consequently, intracellular and extracellular trafficking of haem is regulated to molecular channeling in organelles and ensures detoxification as necessary. *Ho1* mRNA was generally enhanced in the kidney tissues from mice in the current study (Figure 5) although kidney *abcg2* mRNA levels were not significantly different. The kidney is important in iron metabolism for the biogenesis and biodegradation of haem. The former is by a regulatory feedback loop of erythropoietin production and the latter by the glomerullar filtration of haem by megalin and cubilin receptors (Gburek et al., 2002, 2003). Consequently, Abcg2 might not be involved in the efflux of haem/porphyrin in the kidney as it does in erythroid progenitor cells. Perhaps megalin and cubilin function in concert and are complementary with *ho1* to exude the resultant porphyrin breakdown products of haem: this remains an unanswered question.

In conclusion, abcg2, effluxes excess porphyrin metabolites and possibly excess haem during erythropoiesis particularly under hypoxic conditions. Egress and detoxification of excess porphyrin/haem by abcg2 and flvcr to maintain cellular homeostasis is presumably complemented by ho1 expression during the early stages of erythropoiesis. Consequently, abgc2 mRNA levels are stimulated during enhanced erythropoiesis in extramedullary haematopoietic splenic and liver tissues of Hpx, PHZ-treated, hypoxic, and iron-deficient mice. However, abcg2 expression is low in the kidney, and in tissues of this organ ho1 degradation of haem might predominate. Tissue-specific expression of haem influx/efflux proteins and haem catabolism by ho1 must be critically balanced to maintain optimal levels of haem for metabolic processes.

### Conflict of interest statement

The authors declare that the research was conducted in the absence of any commercial or financial relationships that could be construed as a potential conflict of interest.
